# Investigation of Cyprinid Herpesvirus 3 (CyHV-3) Disease Periods and Factors Influencing CyHV-3 Transmission in A Low Stocking Density Infection Trial

**DOI:** 10.3390/ani12010002

**Published:** 2021-12-21

**Authors:** Isaiah E. Tolo, Przemyslaw G. Bajer, Tiffany M. Wolf, Sunil K. Mor, Nicholas B. D. Phelps

**Affiliations:** 1 Minnesota Aquatic Invasive Species Research Center, University of Minnesota, St. Paul, MN 55108, USA; tolo0007@umn.edu (I.E.T.); bajer003@umn.edu (P.G.B.); kumars@umn.edu (S.K.M.); 2 Department of Fisheries, Wildlife, and Conservation Biology, College of Food, Agriculture and Natural Resource Sciences, University of Minnesota, St. Paul, MN 55108, USA; 3 Department of Veterinary Population Medicine and Veterinary Diagnostic Laboratory, College of Veterinary Medicine, University of Minnesota, St. Paul, MN 55108, USA; wolfx305@umn.edu

**Keywords:** cyprinid herpesvirus 3, transmission, transmissibility, contact rate, infection trial, incubation period, symptomatic, disease avoidance, virulence-transmission tradeoffs

## Abstract

**Simple Summary:**

Pathogens are the primary limitation to aquaculture production of fish and a major issue in consideration of the interface between cultured and wild populations of fishes worldwide. While rapid spread of fish pathogens between populations (wild or farmed) is generally anthropogenic and the result of trade, the mechanisms of transmission once a pathogen has been introduced to a fish population are not well understood. The most widespread pathogen impacting both aquaculture and wild populations of common carp (*Cyprinus carpio*, carp) is Cyprinid herpesvirus 3 (CyHV-3). To understand how CyHV-3 is transmitted in a population we conducted a series of infection trials, designed to determine the kinetics CyHV-3 infections, identify the contributions of direct and indirect forms of CyHV-3 transmission, and to determine the contributions of contact rate, viral load, pathogenicity, and contact type. We found that direct contact between fish was the primary mechanism of CyHV-3 transmission rather than transmission through contaminated water. Additionally, CyHV-3 transmission occurred primarily during the incubation period of CyHV-3, prior to the appearance of disease signs and disease-associated reduction in contact rate.

**Abstract:**

Cyprinid herpesvirus 3 (CyHV-3) is the etiological agent of koi herpesvirus disease (KHVD) and important pathogen of aquaculture and wild populations of common carp worldwide. Understanding the relative contributions of direct and indirect transmission of CyHV-3 as well as the factors that drive CyHV-3 transmission can clarify the importance of environmental disease vectors and is valuable for informing disease modeling efforts. To study the mechanisms and factors driving CyHV-3 transmission we conducted infection trials that determined the kinetics of KHVD and the contributions of direct and indirect forms of CyHV-3 transmission, as well as the contributions of contact rate, viral load, pathogenicity and contact type. The incubation period of KHVD was 5.88 + 1.75 days and the symptomatic period was 5.31 + 0.87 days. Direct transmission was determined to be the primary mechanism of CyHV-3 transmission (OR = 25.08, 95%CI = 10.73–99.99, *p* = 4.29 × 10^−^^18^) and transmission primarily occurred during the incubation period of KHVD. Direct transmission decreased in the symptomatic period of disease. Transmissibility of CyHV-3 and indirect transmission increased during the symptomatic period of disease, correlating with increased viral loads. Additionally, potential virulence-transmission tradeoffs and disease avoidance behaviors relevant to CyHV-3 transmission were identified.

## 1. Introduction

Cyprinid herpesvirus 3 (CyHV-3) is the etiological agent of koi herpesvirus disease (KHVD), an exceptionally impactful disease of aquaculture and wild populations of common carp and the ornamental variety, koi (*Cyprinus carpio*, carp) [1]. Outbreaks of KHVD were first reported in koi and farmed carp in the late 1990’s in Germany, Israel and the USA [1,2], but reports of KHVD outbreaks rapidly extended to other European countries as well as South Africa, Indonesia, Taiwan, and Japan by the early 2000’s [3,4,5,6]. Today KHVD is a global epidemic, with a reported distribution covering almost every continent and with mass mortality events occurring in both aquaculture and wild populations of carp [7]. Despite advances in the development of diagnostic tools, prevention strategies and immunizations, morbidity and mortality of farmed and wild carp caused by KHVD still have major impacts (e.g., recent outbreak in Iraq resulting in 100% mortality of a farm containing ~2 million carp) [8]. The economic impact of KHVD in farms and communities experiencing outbreaks can be severe (e.g., ~$15 million USD in estimated losses for Indonesian koi and carp farmers from 2003–2005) [9]. Leveraging the impacts of KHVD, ongoing research is evaluating the potential value of CyHV-3 as a biological control agent for carp in Australia, where invasive populations are ecologically and economically damaging [10]. While controversial, this important consideration of CyHV-3 has led to interest in understanding the impacts of outbreaks of KHVD in wild populations of carp in regions where the virus has become endemic [11,12,13].

CyHV-3 is an enveloped double stranded DNA herpesvirus belonging to the family *Alloherpesviridae*, along with other herpesviruses affecting fish and amphibians [14]. Carp affected by KHVD show clinical signs including lethargy, appetite loss, increased respiratory frequency, pale or necrotic gills, skin lesions, enophthalmia and neurological signs such as erratic swimming and loss of equilibrium [2,15,16,17]. Mortality caused by KHVD is caused by a combination of pathological alterations to the gills and kidney as well as severe skin alterations leading to hypo-osmotic shock [2,18,19]. The marked seasonal occurrence of KHVD outbreaks in farmed and wild populations appears to be related to water temperature, with most outbreaks of KHVD reported when water temperatures are between 18–28 °C [20,21,22]. Experimentally, temperatures of 16–18 °C are also permissive for the development of KHVD in carp though onset of disease is delayed [20,21]. During acute infections at optimal temperatures CyHV-3 may be detected in virtually any tissue as early as 1–2 days post exposure (dpe) [18]. Disease onset is variable but may occur between 2–6 dpe and mortality between 7–27 dpe [13,23]. Carp surviving infection with CyHV-3 may become latent carriers of CyHV-3, which shed the virus after reactivation in response to temperature changes, immune suppression, or other stressors [24,25,26].

In general, viruses of the family *Alloherpesviridae* are host specific, lacking intermediate hosts and infecting only a limited range of hosts [14,27,28]. Though KHVD is limited to *C. carpio* there is evidence that CyHV-3 may be asymptomatically carried by a variety of non-carp fishes which may contribute to the transmission of CyHV-3 between/within farmed and wild populations [29,30,31,32]. Whether asymptomatic carriers act as mechanical vectors of CyHV-3 or can become infected (i.e., with evidence of viral replication) is still under investigation [13,33,34,35]. Regardless of the role of non-carp species, CyHV-3 is easily spread via the movement of infected carp given the delayed onset of disease and the potential for persistent/latent carriers [24,36,37]. The practice of cohabitating koi in the same tanks during exhibitions is a potential explanation for the rapid globalization of CyHV-3 [38]. In wild and farmed populations of carp, the movement of live fish, release of infected koi and transmissions via waterfowl and piscivorous birds have all been implicated as potential routes of dispersal of CyHV-3 [4,39,40]. However, the transmission of CyHV-3 in European carp farms is best explained by the movement of live carp [39,41] and other indirect mechanisms have not been fully demonstrated to occur outside of laboratory conditions.

Once CyHV-3 has spread to a population, transmission is horizontal and thought to occur via both direct and indirect routes [42]. During acute infections, CyHV-3 may be detected in almost all tissues, including the gills, skin and gut which likely represent sources of viral excretion [18,43,44,45]. The skin epidermal tissue (of the face, nasal cavity, fins or pharyngeal epidermis and mucosa) have all been demonstrated as major portals of entry for CyHV-3, which are likely relevant for both direct and indirect transmission [19,46,47,48,49,50]. Gill tissue is a major secondary site of viral replication [44,47], and some studies suggest that early replication of CyHV-3 and pathology in gill tissue indicates that gill may also serve as an entryway for CyHV-3 infection, though this has never been conclusively demonstrated [18,19,43,51].

Direct transmission of CyHV-3 may result from skin to skin contact between infectious and naïve carp, either during breeding, social feeding bouts, or necrophagous behaviors [49,52]. Carps’ natural behavior during breeding events may especially favor CyHV-3 transmission, since during this period multiple male carp must press and nudge against breeding females [53]. The skin of the head and pectoral fin rays of male carp become studded with pearl organs (nuptial tubercles) during the breeding period, which roughen the epidermis and allow for increased close contact between male and female carp [53]. Transmission of CyHV-3 in wild populations of carp in Japan seems to be adapted to this aspect of the carp life cycle, with peak reactivation of persistent/latent infections of CyHV-3 corresponding to the breeding period [37,54]. Thus, carp behavior and physiology during the breeding period may increase the likelihood of transmission of CyHV-3 between infectious and naïve partners. To date, there has been no evidence of vertical transmission of CyHV-3.

Water may facilitate indirect transmission since CyHV-3 has been shown to remain infectious in water for at least four hours at temperatures when viral shedding is observed (i.e., 23–25 °C), and lower water temperatures may favor its persistence [55]. In the absence of hosts, free viral particles of CyHV-3 become quickly inactivated in environmental water samples, likely by microorganisms [56]. In carp populations with previous exposure to CyHV-3 in Japan, CyHV-3 DNA has been detected in high concentrations in lake and river water samples, particularly in locations where pre-spawning aggregations of carp were likely to have occurred [57,58,59]. It is hypothesized that attachment of viral particles to particulate inorganic and organic matter as well as accumulation in plankton may allow CyHV-3 to persist in the aquatic environment [60]. However, these mechanisms of indirect transmission are not well described, and viable CyHV-3 particles have not been isolated from environmental sources.

Beyond the route of pathogen transmission, it is also important to understand what factors influence infection to inform disease management and guide the development of epidemiological models [61,62,63]. Measures of the rate or probability of transmission refer to the efficiency of transfer of a pathogen (i.e., transmissibility) from a single infectious individual to a susceptible individual following contact relevant for transmission to occur. Pathogen transmission in fish populations may be influenced by many factors including, viral load of infectious conspecifics, population demographics and density, climate, the presence of pathogen vectors and reservoirs [61], as well as the behavior of both infectious and naïve hosts [64]. The relative influence of these factors on transmission can be difficult to quantify since they often act in concert. For CyHV-3, the influence of temperature on transmission rate has been well described by laboratory investigations [20,21] and mathematical modeling [65]. Seasonal change in water temperature is the main driver of transmission of CyHV-3 in carp populations since temperature influences the timing of seasonal spawning, the metabolic and immune status of carp, and CyHV-3’s transmissibility within and between hosts [37,55]. However, other factors influencing the transmission of CyHV-3 such as viral load of infectious carp and how clinical signs in infected carp interact with transmission have not been well described.

To better evaluate the mechanisms and factors influencing transmission of CyHV-3 we use a laboratory infection trial to determine the relative contributions of direct and indirect transmission of CyHV-3 and examine the influence of viral load and behavior associated with clinical KHVD on the transmissibility of CyHV-3. Our hypotheses were that CyHV-3 is primarily transmitted by direct contact and direct transmission would be increased by contact rate and viral load.

## 2. Materials and Methods

### 2.1. Fish and Virus

This study was carried out in accordance with the recommendations in the Guide for the Care and Use of Laboratory Animals of the National Institutes of Health. All protocols for sampling, procedures and experimental endpoints involving live fish conducted in this study were approved by the Institutional Animal Care & Use Committee (IACUC), University of Minnesota (St. Paul, MN, USA), under the approval numbers IACUC-1806-36036A and 1808-36276A. Experiments were performed in compliance with the ARRIVE guidelines on animal research [66]. All experiments were conducted in the Minnesota Aquatic Invasive Species Research Center’s Containment Laboratory (MAISRC-CL), in a BSL-2 laboratory. All water used in experiments described below originated from an underground aquifer and all effluent water from experimental tanks was pumped directly into disinfection tanks and treated with sodium hypochlorite solution to maintain a minimum of 0.5 mg/L HOCL concentration corresponding to a target of 2.5–3.0 mg/L free chlorine concentration at a pH range of 8.2–8.3 at room temperature (i.e., 22–24 °C) with a bathing cycle time of 30 min.

A total of 316 naïve juvenile (~1 year in age, avg 40 g, 106 mm in standard length) carp were purchased from Osage Catfisheries (Osage Beach, MO, USA) with a CyHV-3-negative diagnostic certification. All carp were housed in a ~3000 L tank with flow through well water (flow rate = 1 tank volume/h) at 21–24 °C and acclimated for 30 days prior to use in the disease trials at the MAISRC-CL. This temperature was chosen as the optimal temperature for CyHV-3 in terms of transmission, optimal growth in cell culture, and development of high viral loads and severe disease in previous experimental infections [18,20,21] and was used in all experimental enclosures. Unless otherwise specified, carp had 12-hour photoperiods and were fed a diet of commercial feed following the manufacturer’s recommendation of 2% body weight per day (Skretting classic trout, Skretting, Tooele, UT, USA). Moribundity was used as an experimental endpoint for this study. Fish determined to be moribund (i.e., resting in lateral recumbency, unable to maintain orientation, not responding to probing with a net) were immediately removed and euthanized in a solution of 3 mL/L clove oil (90% Eugenol) for 15 min.

Cell culture was performed according to the US Fish and Wildlife Service and American Fisheries Society—Fish Health Section Blue Book (AFS-FHS-2014) using the North American KHV/Elysian/2019 strain cultured on common carp brain cells (CCB), as previously described [13].

### 2.2. Trial 1: Disease Periods Trial

To study the factors influencing viral transmission it was first necessary to determine the duration of disease periods for KHVD in naïve juvenile carp. The disease periods evaluated in this study were the pre-infectious period (i.e., latent period), incubation period, prodromal period, clinical period, and infectious period, which are defined in Table 1 [67,68]. Note that the pre-infectious period of disease is described as the time from exposure to infectiousness and does not refer to latent infections of CyHV-3 occurring in convalescent carp [69].

**Figure 1:**
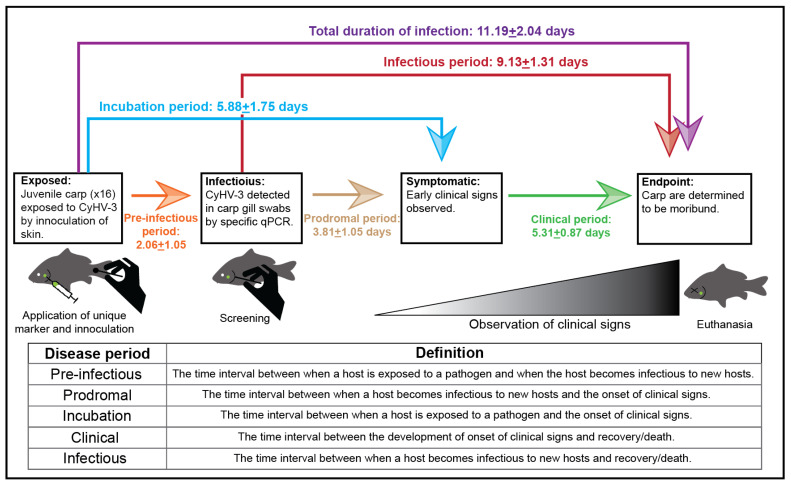
Trial 1 schematic and disease periods. Average values, standard deviations, and definitions are given for each CyHV-3 disease period.

Sixteen carp were anesthetized in a solution of 100 µL/L of clove oil (90% Eugenol; Velona, Elk Grove Village, IL, USA) and inoculated with CyHV-3 via swabbing of the caudal fin/peduncle (three swab strikes across either side of the caudal fin) with CyHV-3 cell culture supernatant (TCID50 = 100/mL, qPCR copy number = 5.89 × 106/mL) using a sterile cotton swab (Dynarex, Orangeburg, NY, USA) (Figure 1). This method of inoculation was chosen to disrupt the mucus layer of carp [52] and to directly inoculate the skin with CyHV-3, allowing us to use gill swabs as evidence of viral shedding and infectiousness. Each fish was uniquely marked with colored injectable elastomer (Northwest Marine Technology, Anacortes, WA, USA) to observe the progression of disease periods in each fish individually, then moved into a 60 L aquarium with flow through well water (flow rate = 3–4 tank volumes/h) at 23 °C.

To identify the timepoints of viral shedding at each day post exposure (1 dpe, 2 dpe, etc.), all fish were collected with a soft net and swabbed with sterile cotton swabs to collect mucus from the gills (i.e., one swab strike across gills, alternating left or right side each day), and then returned to the tank. Swab tips were aseptically broken off into 1.5 mL microcentrifuge tubes (Globe Scientific, Mahwah, NJ, USA) and frozen at –20 °C until nucleic acid extraction and qPCR could be performed (described below). The appearance of early clinical signs (e.g., discolored skin, loss of the mucosal layer, or frayed fins) was noted for each fish and moribund or dead fish were removed from the tank. A control group of sixteen additional carp were inoculated with sterile cell culture medium and sampled and monitored identically to the experimental group to differentiate morbidity and mortality caused by the sampling protocol from the development of KHVD.

### 2.3. Trial 2: Direct and Indirect Contact Trial

**Figure 2:**
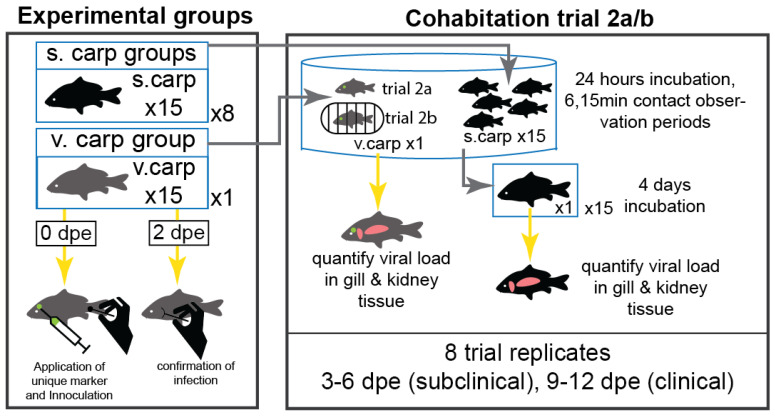
Trial 2a&b schematic. Days post exposure (dpe). Blue lines indicate tank enclosures, numbers within tank enclosures indicate the number of individuals per tank and numbers outside of tank enclosures indicate the number of tanks. Yellow arrows indicate fish movement for inoculation and sampling, and grey arrows indicate movement of fish to new enclosures. Green marks on fish indicate the presence of unique elastomer markings on s.carp. Pink shapes indicate tissue sampling targets of gill and kidney. Vector carp were either cohabitated with sentinel carp in Trial 2a or sequestered in a plastic cage in Trial 2b.

To determine the relative contributions of direct and indirect transmission of CyHV-3, we conducted a two-stage cohabitation trial (Figure 2), allowing carp to have direct physical contact with one another (Trial 2a) or indirect contact where water was shared (Trial 2b).

Trial 2a: Direct contact—Fifteen “virus-exposed carp” (v.carp) were anesthetized in a solution of 100 µL/L of clove oil and inoculated with CyHV-3, uniquely marked and housed as previously described for Trial 1. The v.carp group was screened at two dpe, based on the pre-infectious period determined in Trial 1, by collecting samples of mucus from the gills as described for Trial 1. Any v.carp determined to be CyHV-3 negative were euthanized by immersion in a solution of 3 mL/L pure clove oil for 15 min and discarded. An additional 120 “susceptible carp” (s.carp) were randomly distributed into eight 60 L aquaria (n = 15 s.carp/aquarium). Lastly, 16 “control carp” (c.carp) were mock-inoculated with sterile cell culture medium and uniquely marked for contact counting (below).

To determine the influence of CyHV-3 pathogenicity on transmission, a total of eight serial experiments were done for direct and indirect contact trials; four serial experiments (3–6 v.carp dpe) of the incubation period and four serial experiments (9–12 v.carp dpe) of the clinical period. Time dependency of these serial experiments was used to characterize transmission during the incubation and clinical periods and to observe changes in viral load and transmission during these disease periods. For each serial experiment a single v.carp and group of s.carp (*n* = 15) were placed in ~3000 L cohabitation tank at a flow rate of 10 L/min (~0.2 tank volumes/h) and a temperature of 23 °C. The cohabitation tank had continuous lighting and feed was not administered during the cohabitation period. During each serial experiment, carp were cohabitated for 24 h and direct contact (counts of v.carp contacting any s.carp; ~1 s of direct physical contact = 1 contact) was counted by an observer using a clicker in six, 15 min observation periods. Contact counts were done after a 30 min acclimation period (i.e., after addition of carp to the tank) and were spaced by at least 15 min, with three counts occurring at least 12 h after initiation of the trial. Contacts during each count were categorized as social (normal shoaling behavior observed in control experiments), aggressive (chasing or nipping behavior), or incidental (apparently random contact made by listless swimming behavior of v.carp with late KHVD clinical signs).

Following the cohabitation period, the v.carp was euthanized and frozen at −20 °C until necropsy could be performed. To quantify disease severity, the clinical signs and gross lesions in v.carp were scored from 1 (no visible clinical signs)—4 (severe clinical signs and pathology) based on four categories, including change in skin color, loss of skin tissue, condition of fins, and abnormal behavior (i.e., lethargy, abnormal body orientation), resulting in a maximum score of 16 for severe disease. S.carp were separated into individual static 20 L tanks with aeration at 23 °C for four days then euthanized and frozen at −20 °C until necropsy could be performed. A period of four days was chosen based on the maximum duration of the pre-infectious period determined in Trial 1.

Trial 2b: Indirect contact—To evaluate the influence of indirect contact on CyHV-3 transmission, we repeated the cohabitation trial with an identical study design as described above for Trial 2a. However, to prevent direct contact between “virus exposed carp” (v.carp) and “susceptible carp” (s.carp), the v.carp were sequestered into a double walled plastic cage (present but unused in Trial 2a) once introduced. The cage was composed of a 20 cm diameter, plastic mesh cylinder (10 mm mesh size) placed inside of a larger 30 cm diameter, plastic mesh cylinder. Both cylinders were cut so that they stood 30 cm above the water’s surface to prevent escape of v.carp during the incubation period.

### 2.4. Nucleic Acid Purification and Detection of CyHV-3 by qPCR

All v.carp and s.carp were defrosted and samples of gill and kidney (approximately 100 mg of each tissue) were removed using sterile tools between each fish and tissue type. Tissue samples were homogenized in 1 mL of nuclease-free water (NFW) and then centrifuged, with 50 μL of the resulting supernatant later used as the starting material for nucleic acid purification. The ends of cottons swabs were vortexed in 200 μL of NFW with 50 μL of the resulting supernatant later used as the starting material for nucleic acid purification.

For nucleic acid purification, chelex resin (Sigma) was used as described by Zida et al. (2019) [70]. For each sample type, 150 μL of chilled 80% ETOH was added, then centrifuged and the supernatant removed. Samples were allowed to air dry for 10 min to remove residual ETOH. 150 μL of 20% Chelex was added to each sample and vortexed. Samples were then incubated at 90 °C for 20 min and centrifuged and immediately used for qPCR.

A Taqman probe-based qPCR was used for the detection of CyHV-3 DNA targeting the ORF89 gene [18], using a StepOnePlus thermocycler with default settings (Applied Biosystems). Nucleic acid purifications from all samples were screened for CyHV-3 using a PrimeTime gene expression master mix kit (Integrated DNA Technologies, Coralville, IA, USA), with each reaction containing 400 nM of primers (KHV-86f: GAC-GCC-GGA-GAC-CTT-GTG, KHV-163r: CGG-GTT-GTT-ATT-TTT-GTC-CTT-GTT) and 250 nM of the probe (KHV-109p: [JOE] CTT-CCT-CTG-CTC-GGC-GAG-CAC-G-[IBRQ]. The reaction mix was subjected to an initial denaturation at 95 °C for 3 min, followed by 40 cycles of denaturation at 95 °C for 5 s and annealing at 60 °C for 30 s. A threshold cycle of 38 was used as a cut off. The standard curve for quantification of CyHV-3 genomes was performed using a laboratory synthesized DNA fragment containing the ORF89 sequence as previously described by Padhi et al. (2019) [12]. The results for virus load are presented as the number of viral copies per 50 μL of tissue supernatant.

### 2.5. Statistical Analysis

R 4.0 (R Software, Vienna, Austria) was used for all statistical analyses. The ggplot2 package and Adobe Illustrator (Adobe Inc, San Jose, CA, USA.) were used to prepare figures unless otherwise specified. CyHV-3 qPCR copy numbers were Log transformed prior to all statistical analyses. Descriptions of all continuous and categorical variables measured in this study are provided in Table 2.

To determine the relative contribution of direct and indirect transmission to overall transmission of CyHV-3, the number of secondary infections detected in s.carp in Trial 2a and 2b (i.e., transmission) was compared using odds ratios (OR) and Fisher’s exact tests, computed using the epitab() function from the epitools package [71]. In cases where contingency tables had cells with zero values, 0.5 was automatically added to each cell. Transmission during Trial 2 disease periods (i.e., aggregated data from serial experiments) was also compared using OR’s and Fisher’s exact tests as described above. Transmissibility of CyHV-3 (t) indicates the likelihood of CyHV-3 transmission given physical contacts counted in each trial. The method for calculating transmissibility in each trial are provided in Table 2.

Relationships between all continuous variables measured in Trial 2, were measured using pairwise Pearson correlations, computed using the rcorr() function from the Hmisc package. Correlations were plotted using the corrplot() function from the corrplot package [72]. Prior to measuring correlations, Log CyHV-3 copy numbers were averaged separately for gill and kidney for s.carp in each serial experiment of Trial 2. Contact rates were also averaged for each serial experiment of Trial 2a.

All significant differences in continuous variables measured for Trial 2 experimental groups (i.e., serial experiments, disease periods, and infection groups, including separate tissue types) were determined using 1-way ANOVA with subsequent pairwise multiple comparisons using the Holm-Sidak method, computed using the aov() and pairwise.t.test () functions of the R base stats package.

Regression analysis was done using the lm() function of the R base stats package. To determine the relationship between transmission, contact rate and viral load, multiple regression models were calculated with transmission as the dependent variable and contact rate, viral load, and their interactions as predictors (Table 3). Separate multiple regression models were calculated for each tissue type (i.e., gill and kidney tissues). Multiple regression models (used to determine the relationship of contact rate + viral load to transmission) were compared using AIC, computed using the AIC() function of the R base stats package. Viral load was also analyzed as a predictor of transmissibility and vector pathology scores (i.e., dependent variables).

## 3. Results

### 3.1. Trial 1: Disease Periods Trial

**Figure 3:**
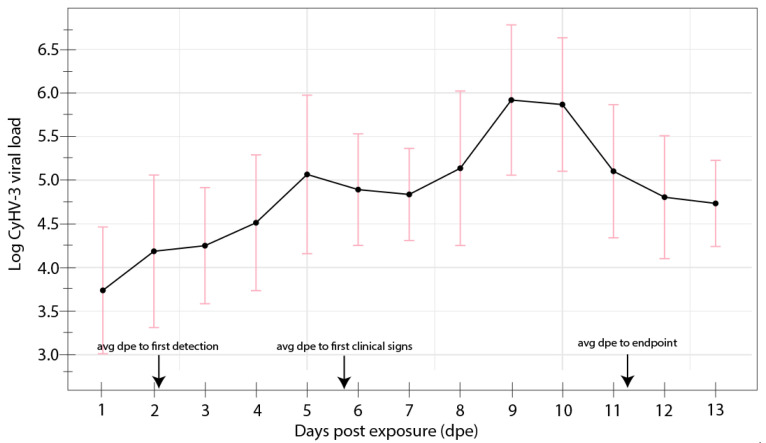
Trial 1 log CyHV-3 copy numbers. Average CyHV-3 viral loads for each day are denoted by points and standard deviations are denoted by pink bars. Average days to first detection of CyHV-3 in gill swabs, to first observation of disease signs and experimental endpoint are denoted with black arrows.

Results of the disease periods experiment are shown in Figure 1. Following the inoculation of carp with infectious cell culture supernatant, CyHV-3 infection and the development of clinical signs consistent with KHVD was confirmed in all 16 carp in the experimental group. CyHV-3 and clinical signs consistent with KHVD were not detected in any carp in the control group though minor darkening of the skin of the caudal peduncle at the inoculation site was observed in five individuals. CyHV-3 DNA was first detected in gill swabs as early as 1 dpe and as late as 4 dpe. The appearance of disease signs occurred in all carp between 4–9 dpe and all fish were determined to be moribund between 8–14 dpe. Early disease signs included loss of the mucosal layer and minor darkening and reddening of the skin as well as minor fraying of fins. Later disease signs, occurring at approximately 9 dpe in most carp, included larger more pronounced skin lesions (exposing underlying muscle tissue in some cases), significant fraying of the fins, blister-like lesions, enopthalmia and behavioral signs such as lack of response to feed, lethargy and resting in lateral recumbency and non-reactiveness to prodding with a net. The average pre-infectious period (i.e., period between exposure and shedding of CyHV-3 detected in gill swabs) was 2.06 + 1.04 days. The average length of the incubation period was 5.88 + 1.75 days with a CyHV-3 log copy no. in gill swabs between 2.91 and 7.40 (mean log copy no.:4.57 + 0.85). The average duration of the clinical period was 5.31 + 0.87 days with the CyHV-3 log copy no. in gill swabs between 3.93 and 7.66, with an average log copy no. of 5.29 + 0.86. The average length of the infectious period was 9.13 + 1.31 days. CyHV-3 viral load in gill swabs increased on average during the infectious period with the highest avg viral loads occurring at 9–10 dpe (Figure 3).

### 3.2. Trial 2: Direct and Indirect Contact Trial

#### 3.2.1. Transmission

**Figure 4:**
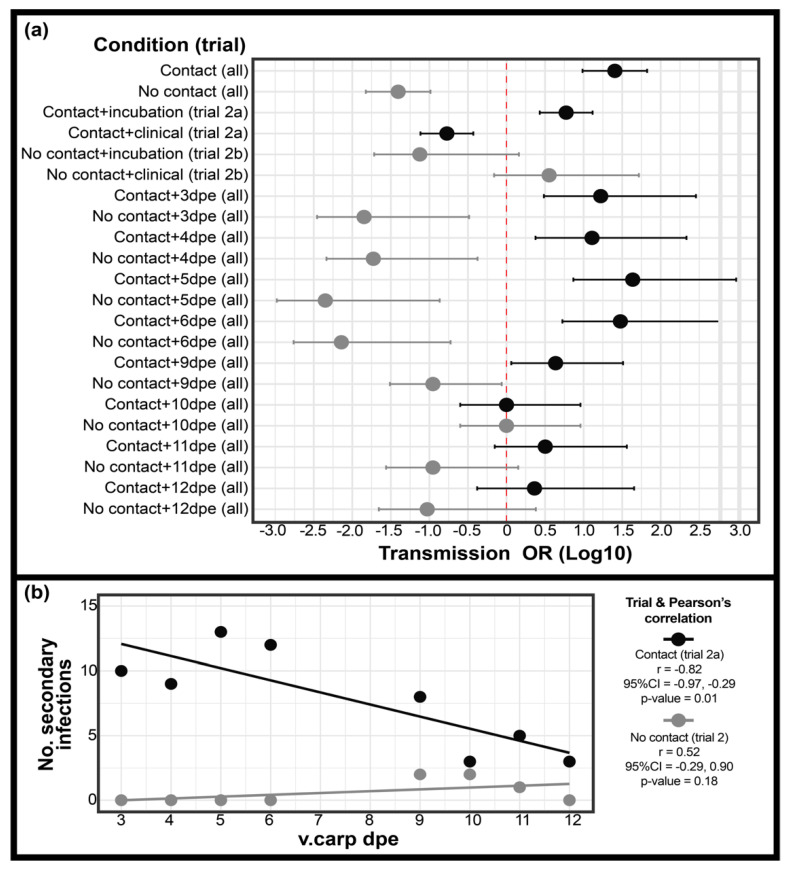
Trial 2 odds of transmission and number of secondary infections. (**a**) forest plot of CyHV-3 transmission odds for contact Trial 2 replicates. Significant ORs are indicated by error error bars that do not cross the center 0 value. “Condition” indicates the contact group (i.e., Trial 2a or 2b) and disease period or experimental replicate for which the OR is calculated. “Trial” indicates which data is considered for comparison and is denoted as“All” (indicates that comparisons are of aggregated data from all other serial experiments) or aggregated Trial 2a or 2b serial experiments only. (**b**) No of secondary infections of s.carp (i.e., transmission) in Trial 2a and 2b.

The odds of transmission were significantly greater under cohabitation conditions allowing for direct contact (Trial 2a) compared to water sharing only with no direct contact (Trial 2b) (OR = 25.08, 95%CI = 10.73–99.99, *p* = 4.29 × 10^−18^) (Figure 4a). In Trial 2a, 63/120 carp were determined to be positive for CyHV-3 while 5/120 of carp were determined to be positive for CyHV-3 in Trial 2b. The odds of transmission were also significantly greater in Trial 2a 3–9 dpe serial experiments relative to Trial 2b, but not for 10–12 dpe serial experiments (Figure 4a). Transmission occurred in each serial experiment of Trial 2a but had a negative correlation with dpe of the infected vector carp (r = −0.82, 95%CI = −0.97, −0.29, *p* = 1.11 × 10^−2^) (Figure 4b). For Trial 2b, transmission only occurred at 9–11dpe and had no significant trend (r = 0.52, 95%CI = 0.29, 0.90, *p* = 0.18). The odds of transmission were also significantly higher during the incubation period relative to the clinical period in Trial 2a (Figure 4a). There was no significant difference in the odds of transmission with consideration of the incubation or clinical period in Trial 2b, though transmission only occurred during the clinical period (Figure 4).

Despite the higher number of secondary infections observed during the incubation period of Trial 2a, the transmissibility of CyHV-3 (t), was higher on average during the clinical period of disease (avg = 2.76 × 10^−1^ + 0.13) compared with the incubation period (avg = 6.33 × 10^−2^ + 0.03) (*p* < 0.05). The t attributable to direct contact transmission was higher than t not attributable to direct contact (i.e., indirect transmission) in every serial experiment of Trial 2a except for 10 dpe (Table 4), however t attributable to direct transmission in the incubation period (average of 3–6 dpe serial experiments = 6.33 × 10^−2^ + 0.03) was not statistically different from t attributable to indirect transmission during the clinical period (average of 9–12 dpe serial experiments = 6.63 × 10^−2^ + 0.05). The t attributable to both direct and indirect transmission was highest at 9 dpe (3.56 × 10^−1^ and 1.19 × 10^−1^ respectively for t attributable to direct and indirect transmission) (Table 4).

#### 3.2.2. Contact Rate

**Figure 5:**
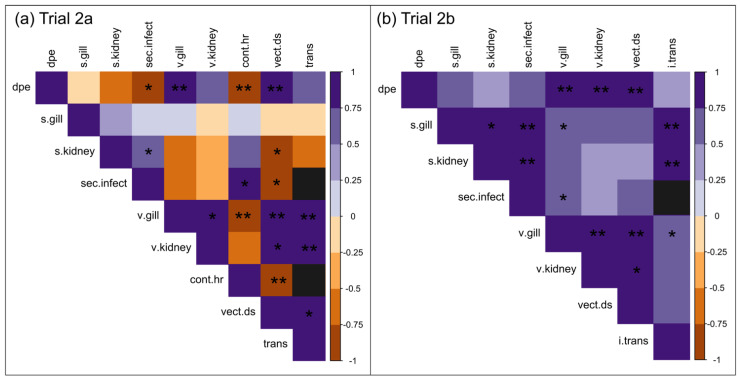
Correlation plots for continuous variables measured in (**a**)Trial 2a/(**b**)Trial 2b. Dpe = days post exposure, s.gill = log viral load in s.carp gill tissue, s.kidney = log viral load in s.carp kidney tissue, sec.infect = number of secondary infections, v.gill = log viral load in v.carp gill tissue, v.kidney = log viral load in v.carp kidney tissue, cont.hr = average number of contacts per hour, vect.ds = vector disease scores, trans = transmissibility, i.trans = transmissibility not attributable to direct transmission (i.e., indirect transmission). Purple shades indicate magnitude of positive correlations. Orange shades indicate magnitude of negative correlations. * = *p* < 0.05, ** = *p* < 0.001.

**Figure 6:**
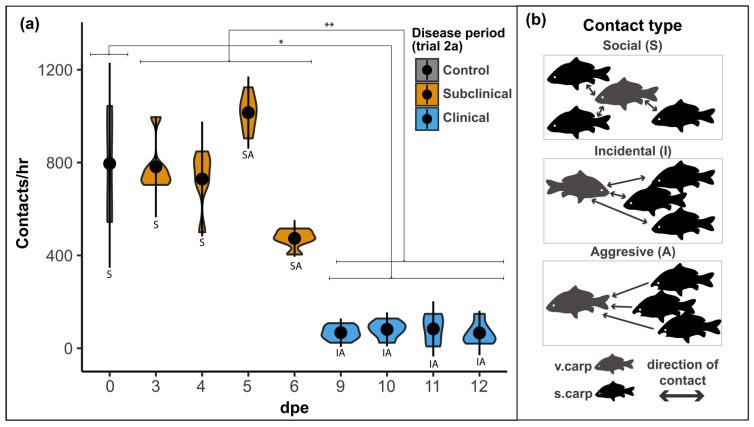
Contact rate and types in Trial 2a. (**a**) violin plot of contact/h for Trial 2a replicates. (**b**) key for contact types observed in trial 2a. * = *p* < 0.05, ** = *p* < 0.001.

Contact rate (i.e., the avg number of contacts observed between v.carp and s.carp per hour during each serial experiment of Trial 2a) had a decreasing trend with dpe in Trial 2a. The contact rate was negatively correlated with v.carp dpe (r = −0.90, *p* = 2.10 × 10^−3^) as well as with v.carp disease scores (r = −0.93, *p* = 6.30 × 10^−4^), and was positively correlated with the number of secondary infections in Trial 2a (r = 0.83 *p* = 1.20 × 10^−2^) (Figure 5a). The contact rate during the incubation period (avg = 750 + 215 contacts) was similar to contact rates for the control group (avg = 767 + 239 contacts) (Figure 6a). The average contact rate of the clinical period (avg = 75 + 43 contacts) was significantly lower than both the average for the incubation period and control groups (Figure 6a). Contact types identified in Trial 2a also changed. The contact type in the control group was always categorized as social (i.e., normal shoaling behavior of v.carp with s.carp). The contact type in Trial 2a was primarily categorized as social during the early-stage incubation period, however, at 5–6 dpe the behavior changed to more social/aggressive (i.e., v.carp shoaled with but was occasionally nipped/chased by s.carp) (Figure 6). Contacts in the Trial 2a clinical period groups were conspicuously different from the control and incubation periods and were categorized as incidental/aggressive, where v.carp avoided s.carp or swam listlessly, not oriented with the s.carp group, and all contacts were the result of nipping/chasing behavior of v.carp by s.carp (Figure 6).

Expressed as minutes of close contact, the average time necessary to transmit CyHV-3 during Trial 2a was 20.61 + 10.22 min of physical contact and ranged between 4.48 min at 9 dpe and 32.41 at 4 dpe with an average of 27.67 + 7.95 min for the incubation period and 6.87 + 3.07 min for the clinical period (Table 4).

#### 3.2.3. Viral Load

Following the direct and indirect transmission contact trial, v.carp and s.carp were screened for CyHV-3 in gill and kidney tissues. CyHV-3 was detected more often in kidney than in gill tissue in s.carp in Trial 2a (62/63 positive in kidney, 26/63 positive in gill tissue). In Trial 2b, CyHV-3 was detected in gill tissue in 4/6 s.carp and in kidney tissue in 3/6 s.carp. Although there was no significant difference in the CyHV-3 viral loads of v.carp tissues when comparing Trial 2a and 2b, the v.carp viral load and transmission rate had a negative correlation coefficient in Trial 2a, while the correlation coefficient was positive in Trial 2b (Figure 5b).

**Figure 7:**
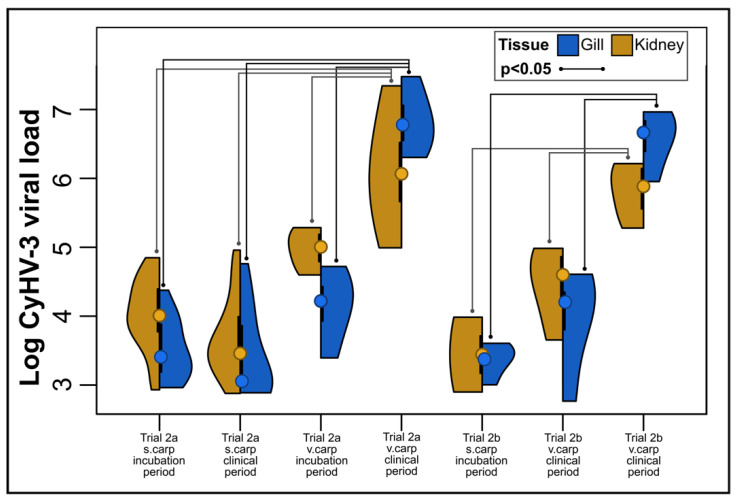
CyHV-3 viral loads in trial 2. Points in split violin plots indicate mean values and black lines indicate standard error. Violin plot areas indicate the distribution of CyHV-3 viral load values. Inset box indicates the tissue type (blue = gill tissue, kidney = kidney tissue) and statistically significant pairwise comparisons.

CyHV-3 viral loads in v.carp gill and kidney tissues in Trials 2a and 2b had an overall trend of increasing with time. With the exception of kidney tissue in Trial 2a, log viral loads of CyHV-3 in v.carp tissues were positively correlated with dpe (Figure 5a). Comparing disease periods, viral loads in v.carp tissues were significantly lower in the incubation period compared to the clinical period (Figure 7). Viral loads of gill and kidney tissues in v.carp did not significantly differ in any serial experiments of Trial 2a/b.

Multiple regression using viral load and contact rate as predictors of transmission rate showed that viral load of both gill and kidney tissue had significant additive effects on transmission rate in Trial 2a (Table 3). In the multiple regression models with an interaction term between viral loads and contact rates, the interaction terms did not have statistical support for either tissue type and AIC values were higher for models with interaction terms than those without, indicating that contact rate and viral load were additive but not multiplicative (Table 3). Viral loads of both tissue types were statistically valid predictors of transmissibility though viral load of kidney tissue was a better predictor of transmissibility in linear regression than viral load of gill tissue (Adjusted r^2 = 0.75 and 0.91 for gill and kidney viral loads respectively) (Table 3).

CyHV-3 viral loads in v.carp tissues in Trial 2a/b also had a positive relationship with increased gross pathology in v.carp, which was quantified using disease scores (Figure 5). Viral loads of either tissue type were statistically valid predictors of disease scores in linear regression, but viral load of gill tissue was a better predictor of disease score than kidney tissue (Adjusted r^2 = 0.85 and 0.50 for gill and kidney viral loads respectively).

## 4. Discussion

In this study we determined the major mechanism of CyHV-3 transmission and quantified the disease periods and transmission dynamics for KHVD in controlled laboratory exposure experiments. We also present a unique dataset, measuring factors that contribute to the transmission of CyHV-3. We simultaneously measured CyHV-3 transmission rates and viral load as well as the contact rate, contact type and disease burden in KHVD infected carp to understand how these multiple factors interact and contribute to CyHV-3 transmission.

The disease periods for KHVD determined in Trial 1 were generally consistent with disease period ranges observed in other studies conducted at similar temperatures. Previous studies show that the pre-infectious period of KHVD is 1–6 dpe at similar temperatures to those used in the present study [19,21,43]. Few studies have attempted to determine duration of the incubation period of KHVD, however, Sunarto et al. (2014) [73] reported observing the earliest gross disease signs occurring earlier between 2 and 5 dpe with behavioral changes (i.e., lethargy, erratic swimming and resting in lateral recumbency) between 5–9 dpe. In the present study we determined the infectious period to range between 6 and 11 days (avg = 9.13 days) which is shorter than that determined by Yuasa et al. (2008) [21], who determined the infectious period to be 12–14 days at 23 °C. The shorter duration of infectiousness in the present study was likely due to the use of moribundity as an experimental endpoint as well as the higher survival of infected carp in the study by Yuasa et al. (2008) [21] (i.e., 30% survival). We also determined that the peak viral load of CyHV-3 in infected carp occurred at 9–10 dpe in Trial 1 gill swabs and gill and kidney tissues from Trial 2. Determination of the kinetics of KHVD in this study was limited to new acute infections in juvenile carp at a single temperature window in preparation for Trial 2, however determining the duration of these disease periods in other temperature ranges and during persistent infections of CyHV-3 in convalescent carp would also be valuable.

Previous studies of CyHV-3 transmission have limited application in natural settings given that they do not fully report stocking densities or have been conducted at unrealistic stocking densities (i.e., 3.5–5 g/L) [21,47,73]. In contrast, we compared the contributions of direct and indirect transmission in a low stocking density transmission trial. Indeed, the optimal stocking density of carp in aquaculture settings is low (i.e., 0.21 g/L) [74] and lower still even for dense populations of wild carp inhabiting natural waterbodies [75]. In some dense populations of carp in Minnesota (USA), carp abundance may reach ~500 kg/ha [75] which can be converted to a density of 1.8 × 10^−6^ g/L using hectare meters as a conversion for hectares of lake area. We used the lowest possible stocking density for our facility without compromising a statistically valid sample size (i.e., serial experiments of 15 s.carp and 1 v.carp stocked at 0.21 g/L) and used a flow-through system to investigate the mechanisms of CyHV-3 transmission that may occur in a natural setting. To our knowledge, this is the lowest stocking density used in a CyHV-3 transmission study and the results of this study provide a reasonable comparison of the mechanisms and drivers of CyHV-3 transmission that occur in low density aquaculture and wild populations of carp. It is important to note however that carp are a social animal and in natural settings, congregate in groups even during summer months [76,77] and aggregate densely during spawning and winter shoaling as well as in artificial feeding sites, thereby making accurate density estimates difficult [11,37,78,79].

We determined that direct contact is the primary mechanism of CyHV-3 transmission. We also found that direct transmission occurred during both disease periods (i.e., incubation and clinical) while indirect transmission only occurred during the later clinical period of disease in Trial 2b. The importance of direct transmission was also found by Boutier et al. (2015) [42], where transmission of a luciferase producing CyHV-3 mutant was higher on average at six and ten dpe in a direct transmission trial compared with an indirect transmission trial. Our findings are also consistent with the prevailing hypothesis of CyHV-3 transmission in natural settings, namely, that transmission occurs through the skin and that transmission may be facilitated via skin-to-skin contact which is more common during breeding [37,54]. The skin of carp has been proposed as the main entryway for CyHV-3 [45]; however, this hypothesis has been questioned due to the anti-viral barrier of carps’ mucosal layer [80]. Indeed, the mucosal layer of carp has been demonstrated to inactivate CyHV-3, and removal of the mucosal layer has been shown to allow for more efficient infection via immersion [52,81]. It may therefore be necessary for the mucosal layer to be disrupted and/or contact to occur where the mucosal layer is thin (e.g., edge of fins) to facilitate CyHV-3 entry through the skin [52,81]. Anecdotally, we observed that the majority of contacts between v.carp and s.carp occurred when the edges of the fins of shoaling carp briefly but repeatedly touched. Though we did not investigate the specific mechanism of direct transmission in this study, our results support the hypothesis that direct contact between infected and naive carp is the primary form of CyHV-3 transmission. Future research could also determine to what extent direct contact increases during breeding and what impact this may have on direct transmission of CyHV-3.

Though indirect transmission occurred much less frequently than direct transmission in Trial 2, the odds of direct transmission at 10–12 dpe were not significantly higher than those of indirect transmission. In this time period, v.carp were clinically ill and contact rate was decreased but transmissibility was high. Importantly, transmissibility attributable to indirect transmission during the clinical period of disease was the same as transmissibility attributable to direct contact during the incubation period of KHVD. Indirect transmission may be particularly important in the aquaculture setting, where populations are held captive and exposed to re-circulating water. This risk has been previously illustrated for CyHV-3 where CyHV-3 DNA has been found on the filters of tanks with infected carp [13] and CyHV-3 transmission has been inhibited after disinfection of water in recirculating aquaculture systems [82].

This study demonstrates that CyHV-3 viral load is an additive factor for both direct and indirect transmission of CyHV-3. Though viral load initially appeared to be negatively correlated with transmission in Trial 2a, the true importance of viral load in increasing transmissibility was identified when we controlled for the significant reduction in contact during the clinical period of disease. The contribution of viral load to transmission of CyHV-3 has not been previously described though CyHV-3 dose administered via immersion in one study did not have a significant impact on transmission [20]. However, viral load has been shown to be positively correlated with the transmission of other viral pathogens of fish such as viral hemorrhagic septicemia and infectious hematopoietic necrosis virus [83,84]. Additionally, viral load is a factor influencing transmission of human viruses that rely on direct forms of transmission such as Herpes simplex virus-2 and influenza virus [85,86]. Interestingly, viral load may also have a nonlinear relationship with transmission [87]. In the case of human viruses such as hand-foot-and-mouth disease and influenza viruses, high viral load has a negative relationship with the duration of the infectious period, which may decrease the number of secondary cases generated by each infectious case [88,89]. The nonlinear relationship between viral load and transmission is also illustrated by virulence trade-off studies of human immunodeficiency virus in which population level declines in set point viral loads correspond with increased transmission opportunity [90,91].

The counterintuitive relationship between CyHV-3 viral load (as well as factors: days post exposure and v.carp disease scores) and overall probability of transmission in Trial 2a is explained by the higher contact rate during the incubation period of KHVD. Indeed, v.carp without clinical signs participated in normal shoaling behavior which brought the infectious and naïve carp into close proximity for long periods of time and allowed for increased contact rate. This behavior transitioned sharply during the clinical period of infection where s.carp were aggressive or only had incidental contact with v.carp.

Shoaling in fish has a dynamic relationship with transmission of fish pathogens. While social shoaling has been implicated as a driver of disease in some cases [92,93], it has also been demonstrated as a mechanism of disease avoidance for individual fish in large shoaling groups [94]. Shoaling behavior of fish has also been shown to become disrupted by parasite infections that impair sensory/motor systems [92,95]. Though we did not determine the mechanism causing v.carp to cease shoaling with s.carp in the clinical period of KHVD in this study, it is well known that infection with CyHV-3 is associated with neuro-degenerative pathology in infected carp [43,48], and that neurological degeneration may disrupt fish shoaling behavior [96].

CyHV-3-infected carp display neurological pathology in the late stage of KHVD [74] and show signs of congestion of the capillaries in brain tissue as well as disassociation of the nerve fibers with the valva cerebelli and medulla oblongata [48], brain centers responsible for postural equilibrium, respiration, and a wide variety of other sensory and motor functions [97,98]. Neurological signs observed in CyHV-3-infected v.carp in this study were similar to those of previous studies [42] and included resting in lateral recumbency, inability to maintain orientation, and general lethargy and listless behavior. Additional changes in the behavior of carp infected with CyHV-3 have been demonstrated by Rakus et al. (2017), who showed that CyHV-3 infected carp move to warmer water in response to viral infection (i.e., behavioral fever). Interestingly, production of a CyHV-3-encoded decoy TNF-a receptor causes a delay in behavioral fever in CyHV-3-infected carp, potentially increasing CyHV-3 transmission by delaying removal of infectious individuals from the population as they seek out thermal refuges [99]. Fish suffering from disease associated behavioral changes may also have an increased susceptibility for removal from the population via predation [95,100], being washed downstream [101], or becoming isolated from groups that are feeding or participating in social behaviors [92].

The disadvantage posed by clinical signs of disease (e.g., virulence) is the basis of the virulence-transmission trade-off hypothesis, which can be used to explain why the transmission of some pathogens is largely dependent on the occurrence of nonclinical disease states. The importance of nonclinical disease-state transmission has been previously described for other viruses such as Epstein Barr virus (EBV), Severe acute respiratory coronavirus 2 (SARS-CoV-2), and Ebola virus [102,103,104,105]. In the case of EBV and SARS-CoV-2, it is possible that most of the transmission may occur during the incubation period of disease when viral loads peak, but infected individuals are nonclinical [102,104]. Likewise, the importance of the transmission of CyHV-3 by nonclinical persistent carriers of CyHV-3 has been previously described [37,65]. Survivors of acute infections with CyHV-3 develop non-replicating CyHV-3 infections that can become reactivated by temperature changes or stress responses [24]. Seasonal reactivation of CyHV-3 infections has been demonstrated to be the basis of endemic CyHV-3 in wild carp in Japan [37], and nonclinical transmission of CyHV-3 by infected ornamental koi may be the cause of its rapid global spread [9]. However, this study is the first to describe the importance of the incubation period of KHVD for CyHV-3 transmission during acute disease. Though CyHV-3 viral loads and transmissibility peaked during the clinical period of this study, v.carp behavior was demonstrated to be an important factor driving transmission during the incubation period of KHVD.

Finally, we also determined that the decrease in contact rate during the clinical period was accompanied by a conspicuous change in the type of contacts occurring between v.carp and s.carp. Contacts were categorized as social during the incubation period of disease as contact was identical to normal shoaling behavior observed in the control trial. However, during the clinical period of KHVD, s.carp became aggressive towards clinical v.carp once behavioral clinical signs became apparent. Social contact is an important driver of diseases in fishes and changes in social behaviors may act as barriers to disease transmission [65,92,93,106]. Modification of fish behavior has mostly been documented for changes in the behavior of diseased conspecifics as described above, but documentation of changes in the interaction between uninfected fish with infected conspecifics (i.e., disease avoidance) is not as common [64,92]. Examples of this type of behavioral change have been documented in mate choice by female three spined sticklebacks (*Gasterosteus aculeatus*), which have been shown to avoid parasite-infected males with impaired sexual ornamentation [107]. Three spined sticklebacks have also been demonstrated to avoid shoaling with individuals infected with visually conspicuous parasites such as microsporidians (*Glugea* sp.) and crustaceans (*Argulus* sp.) [108,109], potentially indicating behavioral avoidance of pathogens based on visual responses. Kiesecker et al. (1999), demonstrated that healthy bullfrog tadpoles (*Rana catesbieana*) were able to detect chemical signals of infection in conspecifics infected with a fungal pathogen (*Candida humicola*) and avoidance behavior was identified in healthy tadpoles and not in infected tadpoles. Interestingly, while tadpoles could express avoidance behavior based on chemical cues released from infected tadpoles, they could not do so when limited to visual cues alone [110]. It has also been demonstrated that attack by penetrating parasites (*Diplostomum* sp.) initiates the release of alarm substances by juvenile rainbow trout (*Oncorhynchus mykiss*), though it is unknown if this response limits transmission [111]. In the present study, aggression displayed by s.carp towards v.carp appeared to occur in response to behavioral changes by v.carp and it is likely that this represents a form of disease avoidance, but future studies should focus on determining the potential visual and chemical cues that may initiate this interesting behavior.

## 5. Conclusions

In conclusion, this study demonstrates that transmission of CyHV-3 during acute infections is driven primarily by direct social contact during the incubation period and increased transmissibility associated with increasing viral loads. Additionally, most CyHV-3 transmission occurs in the incubation period of acute KHVD because of behavioral changes of both infected and uninfected conspecifics associated with behavioral signs of disease in late KHVD. In some aquaculture populations of carp it is also likely that indirect transmission is an important mechanism for the spread of CyHV-3. These findings should be considered in future disease modeling efforts for CyHV-3 since they can guide foundational modeling decisions and improved models that consider within-host disease dynamics. Additionally, disease prevention and management will also benefit from an understanding of the major drivers of early epidemic spread. Finally, additional effort is needed to determine the mechanism by which CyHV-3 is transmitted by direct contact, the pathological changes in infected carp that precipitate behavioral changes, as well as the cues by which uninfected carp respond to clinical KHVD in infected conspecifics.

## Figures and Tables

**Table 1:** CyHV-3 disease periods.

CyHV-3 Disease Period.	Definition	Experimental Approach
Pre-infectious	Time period between when a host is exposed to a pathogen and when the host becomes infectious to other hosts.	Determined by measuring the time between inoculation of carp with CyHV-3 and viral detection in gill swabs.
Incubation period	Time period from pathogen exposure to onset of clinical disease signs.	Determined by measuring the time between inoculation of carp with CyHV-3 and observation of clinical signs.
Prodromal period	Time period between when a host becomes infectious and prior to the development of clinical signs.	Determined by subtracting the pre-infectious period from the incubation period.
Clinical period	Time period during which clinical signs are observed.	Determined by measuring the time between first observation of clinical signs of KHVD and experimental endpoints.
Infectious period	Time period in which the host can infect another host or vector.	Determined by measuring the time between first detection of CyHV-3 in gill swabs and experimental endpoints.

Disease periods definitions were obtained from Thomas et al. (2001) and Mueller et al. (2008).

**Table 2:** Variables measured in this study.

Variable Name.	Variable Type	Variable Description *
Days post exposure (dpe)	Continuous	Indicates either days post inoculation via skin swabbing or days post cohabitation with infected v.carp.
Viral load	Continuous	Log copy number or average log copy number of CyHV-3 genome copies per 50 μL of tissue supernatant measured by specific qPCR.
Transmission	Continuous	Proportion of s.carp determined to be positive for CyHV-3 after 4 dpe.
Contact rate	Continuous	Average (avg) number of brief contacts between v.carp and s.carp expressed as contacts/h (avg 15 min count × 4).
Vector disease score	Continuous	A score based on 4 categories of pathological signs scored on 1:4 point scale, yielding a 1:16 points indicating the level of gross pathology observed in v.carp.
Transmissibility (*t*)	Continuous	Likelihood of CyHV-3 transmission given physical contacts in Trial 2a, calculated for each trial replicate of Trial 2a/b as: *t =* transmission(Trial 2a)/avg contacts (Trial 2a)
		(1) *t* attributable to direct contact = transmission (Trial 2a)/avg contacts (Trial 2a)—transmission (Trial 2b)/avg contacts (Trial 2a)
		(2) *t* not attributable to direct contact = transmission (Trial 2b)/avg contacts (Trial 2a)
Contact type	Categorical	Categorization of contacts between v.carp and s.carp (Trial 2a) as social, incidental, or aggressive.

* “virus exposed carp” (v.carp), “susceptible carp” (s.carp).

**Table 3:** Multiple regression analysis of viral load and contact rate as predictors of transmission, and regression analysis of viral load as a predictor of transmissibility and vector disease scores.

Dependent Variable	Predictor(s)	Interaction	Coefficients				Adjusted r^2	*p*-Value	AIC
			Name	Estimate	*SE*	*p*-Value			
Transmission	Viral load (gill), contact rate	Yes	Intercept	−56.09	33.64	1.71 × 10^−1^	0.80	2.50 × 10^−2^	36.12
			Log viral load (gill)	8.16	5.42	2.07 × 10^−1^			
			Log contacts/h	4.91	5.36	4.11 × 10^−1^			
			Interaction	−0.26	0.97	8.02 × 10^−1^			
Transmission	Viral load (kidney), contact rate	Yes	Intercept	−9.57	32.51	7.83 × 10^−1^	0.81	2.10 × 10^−2^	35.55
			Log viral load (kidney)	−0.14	7.17	9.85 × 10^−1^			
			Log contacts/h	−1.09	6.32	8.72 × 10^−1^			
			Interaction	0.82	1.43	5.96 × 10^−1^			
Transmission	Viral load (gill), contact rate	No	Intercept	−48.45	16.19	3.00 × 10^−2^	0.83	5.00 × 10^−3^	34.26
			log viral load (gill)	6.79	1.65	9.00 × 10^−3^			
			log contacts/h	3.53	1.34	4.70 × 10^−2^			
Transmission	Viral load (kidney), contact rate	No	Intercept	−27.54	8.37	2.20 × 10^−2^	0.83	5.00 × 10^−3^	34.19
			log viral load (kidney)	3.96	0.69	2.00 × 10^−3^			
			log contacts/h	2.5	0.94	4.60 × 10^−2^			
Transmissibility	Viral load (gill)	Na	Intercept	−0.30	0.11	3.11 × 10^−2^	0.74	3.90 × 10^−3^	14.98
			Log viral load (gill)	0.09	0.02	3.90 × 10^−3^			
Transmissibility	Viral load (kidney)	Na	Intercept	−0.69	0.10	4.77 × 10^−4^	0.91	1.34 × 10^−4^	23.79
			Log viral load (kidney)	0.16	0.02	1.34 × 10^−4^			
Vector disease score	Viral load (gill)	Na	Intercept	−12.99	3.38	8.50 × 10^−3^	0.85	6.97 × 10^−4^	40.40
			Log viral load (gill)	3.80	0.60	6.97 × 10^−4^			
Vector disease score	Viral load (kidney)	Na	Intercept	−20.78	10.28	8.98 × 10^−2^	0.50	3.04 × 10^−2^	50.07
			Log viral load (kidney)	5.16	1.83	3.04 × 10^−2^			

**Table 4:** Transmission, contact rate and transmissibility CyHV-3 attributable to direct and indirect transmission.

	Trial 2a/b Serial Experiments (dpe)									
Value	3	4	5	6	9	10	11	12	3–6 dpe avg (SD)	9–12 dpe avg (SD)
Transmission (Trial 2a)	10	9	13	12	8	3	5	4	11.00 (1.83)	5.00 (2.16)
Transmission (Trial 2b)	0	0	0	0	2	2	1	0	0.00 (0.00)	1.25 (0.96)
Avg contacts (15 min count period)	195.50	182.33	253.83	118.33	16.83	20.33	20.83	16.50	187.50 (55.60)	18.63 (2.27)
Min contact for transmission	31.28	32.41	31.24	15.78	4.49	32.53	8.33	6.60	27.67 (7.95)	6.87 (3.07)
Transmissibility (*t*)	5.12 × 10^−^^2^	4.94 × 10^−^^2^	5.12 × 10^−^^2^	1.01 × 10^−1^	4.75 × 10^−1^	1.48 × 10^−1^	2.40 × 10^−1^	2.42 × 10^−1^	6.33 × 10^−2^ (0.03)	2.76 × 10^−1^ (0.14)
*t* attributable to direct contact (direct transmission)	5.12 × 10^−2^	4.94 × 10^−2^	5.12 × 10^−2^	1.01 × 10^−1^	3.56 × 10^−1^	4.92 × 10^−2^	1.92 × 10^−1^	2.42 × 10^−1^	6.33 × 10^−2^ (0.03)	2.10 × 10^−1^ (0.13)
*t* not attributable to direct contact (indirect transmission)	0.00	0.00	0.00	0.00	1.19 × 10^−1^	9.84 × 10^−2^	4.80 × 10^−2^	0.00	0.00 (0.00)	6.63 × 10^−2^ (0.05)

## Data Availability

The datasets generated and/or analyzed during the study are available in the Data Repository for the University of Minnesota (https://conservancy.umn.edu/handle/11299/225571).
